# X-ray Structure-Based Chemoinformatic Analysis Identifies Promiscuous Ligands Binding to Proteins from Different Classes with Varying Shapes

**DOI:** 10.3390/ijms21113782

**Published:** 2020-05-27

**Authors:** Christian Feldmann, Jürgen Bajorath

**Affiliations:** Department of Life Science Informatics, B-IT, LIMES Program Unit Chemical Biology and Medicinal Chemistry, Rheinische Friedrich-Wilhelms-Universität, Endenicher Allee 19c, D-53115 Bonn, Germany; cfeldmann@bit.uni-bonn.de

**Keywords:** small molecules, multitarget activity, promiscuity, protein classes, complex X-ray structures, activity data, binding modes, shape similarity

## Abstract

(1) Background: Compounds with multitarget activity are of interest in basic research to explore molecular foundations of promiscuous binding and in drug discovery as agents eliciting polypharmacological effects. Our study has aimed to systematically identify compounds that form complexes with proteins from distinct classes and compare their bioactive conformations and molecular properties. (2) Methods: A large-scale computational investigation was carried out that combined the analysis of complex X-ray structures, ligand binding modes, compound activity data, and various molecular properties. (3) Results: A total of 515 ligands with multitarget activity were identified that included 70 organic compounds binding to proteins from different classes. These multiclass ligands (MCLs) were often flexible and surprisingly hydrophilic. Moreover, they displayed a wide spectrum of binding modes. In different target structure environments, binding shapes of MCLs were often similar, but also distinct. (4) Conclusions: Combined structural and activity data analysis identified compounds with activity against proteins with distinct structures and functions. MCLs were found to have greatly varying shape similarity when binding to different protein classes. Hence, there were no apparent canonical binding shapes indicating multitarget activity. Rather, conformational versatility characterized MCL binding.

## 1. Introduction

Small molecules with activity against multiple targets, also termed promiscuous compounds [1], are of increasing interest in pharmaceutical research because they are able to elicit polypharmacological effects that often contribute strongly to the efficacy of drugs [2,3,4,5]. Rationalizing multitarget activity of small molecules is equally scientifically stimulating and relevant for practical applications. An ultimate goal of understanding the molecular foundations of promiscuity is translating such insights into the design of novel ligands with pre-defined multitarget activity [5,6,7]. However, the assessment of compound promiscuity is complicated by potential experimental artifacts and varying activity data confidence [8,9,10]. Several data analysis and data mining approaches have been introduced to identify or predict promiscuous compounds [1,11,12,13]. Clearly, the most reliable approach to confirm multitarget activity of small molecules and explore binding events at the molecular level of detail is analyzing and comparing three-dimensional structures of ligand-target complexes [14,15]. It has been shown, for example, that promiscuous compounds often bind to similar protein domains and binding sites [15,16,17], as one might anticipate. On the other hand, ~700 crystallographic ligands were identified that formed complexes with proteins from different families [18]. Multifamily ligands were chemically diverse (>98% non-analog ligands) and frequently displayed similar binding modes interacting with different proteins (median root mean square deviation (RMSD): 1 Å). However, they typically formed different interaction hotspots in binding sites [19]. Taken together, these findings provided some initial insights into promiscuous binding events. 

To further explore multitarget activity of compounds at the molecular level, we have been interested in carrying out a systematic analysis of structural and activity data focusing on binding shapes of compounds in different protein environments in combination with molecular property analysis. The results of our analysis are presented in the following section.

## 2. Results

### 2.1. Target Classification

Emphasis was placed on studying compound binding to distantly or unrelated proteins, given that evolutionarily defined protein families have varying degrees of relatedness and may contain similar ligand binding domains and binding sites. Therefore, in this study, small molecule targets were assigned to functional protein classes following Gene Ontology (GO) [20]. GO classes combine functionally related families, and members of different classes are thus more distant from each other than members of more narrowly defined families. Therefore, we have focused our current analysis on multiclass rather than multifamily ligands.

### 2.2. X-ray Structure-Based Identification of Single- and Multi-Class Ligands

**Figure 1:**
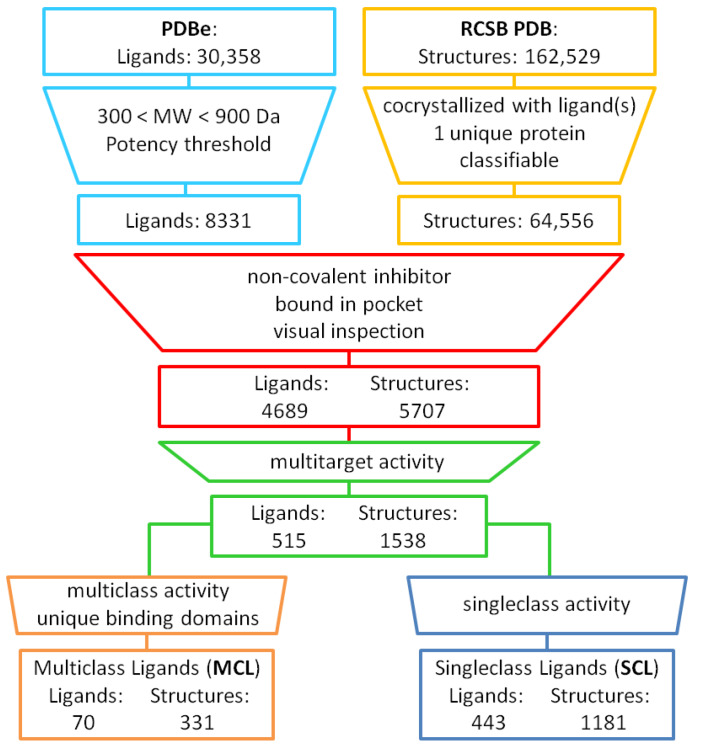
Large-scale data analysis scheme. Ligands from X-ray structures were filtered for criteria such as molecular weight, minimum potency, and (bio) chemical characteristics. X-ray structures of classified proteins in complex with qualifying ligands were analyzed for non-covalent binding events. Ligands binding to multiple targets from the same protein class (single-class ligands, SCLs) and from different classes (multiclass ligands, MCLs) were identified. Two of 72 originally assigned MCLs were found to bind exclusively to very similar domains in different proteins and were thus not further analyzed.

Data sources and selection criteria for our analysis are detailed in the Materials and Methods Section. Figure 1 below summarizes the analysis concept and distinguishes different stages. From the Protein Data Bank (PDB) [21], X-ray structures of protein complexes with small molecular ligands were extracted, provided the target could be classified on the basis of GO. Accordingly, a total of 64,556 complex structures were obtained, including 17,006 unique proteins from 20 different GO classes. These structures were considered for further analysis if they contained one of 8331 ligands for which a potency annotation of at least 10 µM was available. Applying a threshold for at least weak compound potency was considered important to avoid potential overestimation of specific binding events on the basis of X-ray structures, taking typically high local compound concentration under co-crystallization conditions into account. Furthermore, only non-covalent inhibitors were considered and ligands such as peptides, saccharides, various nucleosides, or other compounds not relevant for our chemoinformatics-oriented analysis were omitted, which reduced the number of qualifying complex structures and crystallographic ligands to 5707 and 4689, respectively. A subset of 1538 complexes yielded 515 ligands (11%) with structurally confirmed multitarget activity (without requiring a potency annotation, nearly 1500 ligands would have been obtained). The distribution of protein structures over ligands is reported in Supplementary Figure S1. The 515 promiscuous compounds contained 443 single-class ligands (SCLs) from 1181 complex structures and 70 multiclass ligands (MCLs) covering 331 structures. As an additional criterion, all MCLs were required to bind to structurally distinct protein domains. The 70 MCLs we identified provided the basis for our subsequent analysis. All 70 MCLs are listed with their PDB identifier and the number of associated proteins and protein classes in Supplementary Table S1. Ligands binding to multiple distantly related or unrelated targets are expected to be rare, consistent with our findings. Since our analysis has been comprehensive, one can anticipate similar rates of MCLs among newly identified X-ray ligands with confirmed multitarget activities. There are no intrinsic limitations in interpreting these findings.

### 2.3. Distribution of Multiclass Ligands

**Figure 2:**
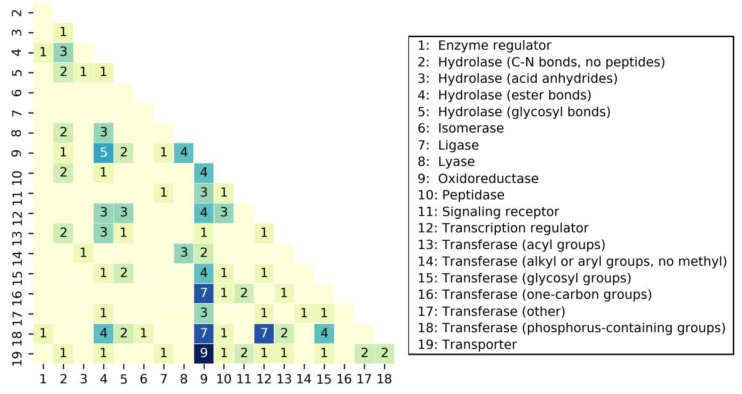
Shared MCLs. The heatmap shows the number of MCLs shared by different protein classes.

Table 1 reports the distribution of MCLs over different protein classes. The corresponding distribution of SCLs over different protein classes is reported in Supplementary Table S2. The 70 MCLs were bound to a total of 255 unique targets from 19 different classes. The number of complex structures per class varied significantly, with only a single complex available for the enzyme regulator and isomerase class and a maximum of 81 complexes for the oxidoreductase class. The protein classes covered by MCLs mostly consisted of enzymes and included six different classes of transferases and four different classes of hydrolases having distinct functions. In addition to enzymes, signaling receptors, transcription regulators, and transporter proteins were also present. With 36 compounds, the oxidoreductase class was associated with most MCLs, followed by one of the transferase classes with 18, and the transporter class with 15 MCLs. Figure 2 shows the distribution of MCLs over pairs of protein classes. MCLs were widely distributed across different pairs, including the oxidoreductase class that shared varying numbers of MCLs with 15 other classes. There were no strongly preferred pairs of classes. The largest number of shared MCLs was nine for the oxidoreductase and transporter class. Hence, the distribution of MCLs over different target classes was overall balanced.

### 2.4. Comparison of Ligand Binding Modes

**Figure 3:**
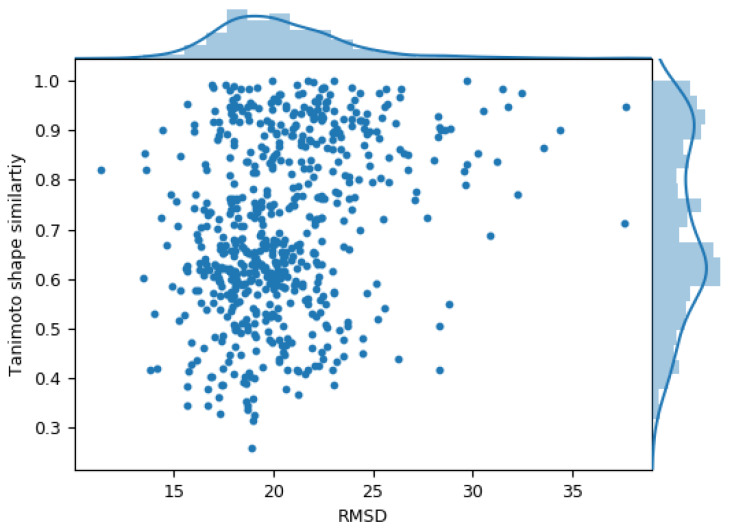
MCL shape similarity versus target RMSD. The scatter plot reports pairwise comparisons of MCL complexes containing proteins from different classes. Each dot represents a pair of complex structures. The shape similarity of the bound conformations of the MCL in both complexes is plotted against the alpha carbon RMSD of the protein structures on the basis of best possible rigid body superposition. In addition, the value distributions along the horizontal and vertical axes are represented as histograms.

Next, we investigated a central question of our analysis by comparing the binding modes of MCLs in proteins from different classes. Figure 3 shows the distribution of Tanimoto shape similarity values for pairwise comparisons of complexes plotted against the RMSD (Å) of best superimposed alpha carbon traces of the target proteins. The large RMSD values for compared proteins reflected the presence of distinct structures. For the calculation of shape similarity, MCL binding modes were optimally superimposed (see the Materials and Methods Section). Importantly, shape similarity values were very widely distributed, ranging from ~0.3, indicating very low shape similarity, to values close to 1 for nearly identical binding shapes. Thus, in some instances, MCLs adopted essentially the same binding mode in different proteins and in others, they bound with distinct conformations. Many intermediate shape similarity values were obtained that also indicated the presence of binding mode variations in different protein environments. The Tanimoto shape similarity value distribution is bimodal in nature. We analyzed the distribution of rotatable bonds of MCLs in compared X-ray structures (Supplementary Figure S2) and identified overall largest subsets of shared MCLs having 0–2 and 4–11 rotatable bonds, respectively. These subsets of MCLs were likely to cause the bimodal distribution of shape similarity values based upon pairwise comparisons. Extending the analysis of protein similarity beyond sequence-dependent methods, binding site similarity scores were calculated for pairs of complexes and plotted against ligand shape similarity (Supplementary Figure S3). There was no apparent relationship between these protein and ligand similarity measures.

**Figure 4:**
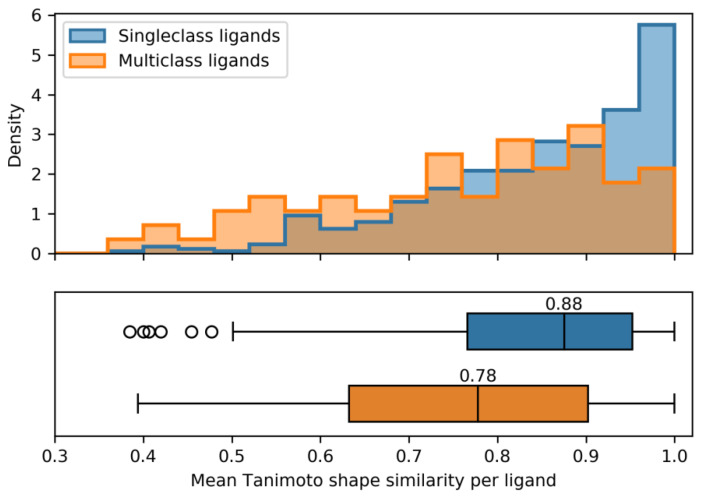
Shape similarity of MCLs versus SCLs. Histograms (top) and boxplots (bottom) report the distribution of shape similarity values of MCLs and SCLs on the same scale. For each ligand, shape similarity was calculated as mean of pairwise comparisons of binding modes across all targets. Boxplots show the lower quartile (left boundary of the box), median value (vertical line), and upper quartile (right boundary of the box). Additionally, whiskers indicate the 1.5-fold of the interquartile range. Statistical outliers are depicted as circles.

In addition, Figure 4 shows the comparison of mean shape similarity for MCLs and SCLs. As revealed by both the histograms and boxplots, SCLs displayed a clear tendency of binding to their targets (from the same class) with binding modes having higher shape similarity than those of MCLs, as one might expect (Mann–Whitney U test, *p*-value $8.1\cdot 10^{-6}$). For MCLs, the distribution of mean shape similarity values was much wider than for SCLs, with median value of 0.78 and 0.88, respectively. These observations also reflected further increased binding mode variability among MCLs relative to SCLs.

### 2.5. Molecular Properties

**Figure 5:**
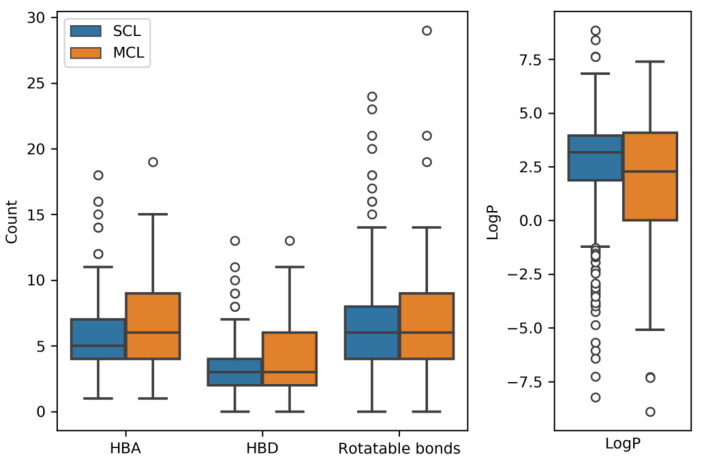
Molecular properties of SCLs and MCLs. Boxplots report the distributions of the number of hydrogen bond acceptors (HBA), hydrogen bond donors (HBD), and rotatable bonds, as well as of the logarithm of the octanol-water partition coefficient (LogP).

We also compared the distribution of hydrogen bond donor and acceptor functions in MCLs and SCLs as well as the number of rotatable bonds per ligand (as a measure of flexibility) and LogP values (as a measure of hydrophobic character). The results are shown in Figure 5. For both sets of ligands, the distributions of descriptors were compared using the Mann–Whitney U test since the descriptors were not normally distributed. The distributions of rotatable bonds were very similar in both cases (*p*-value = 0.45). With median values of six rotatable bonds per compound, many MCLs and SCLs were intrinsically flexible. MCLs were found to contain overall more hydrogen bond donors (*p*-value = $5.4\cdot 10^{-3}$) and acceptors (*p*-value = $4.7\cdot 10^{-2}$) than SCLs, which was an interesting observation considering the variable binding modes of MCLs discussed above. In complexes, donor and acceptor functions are typically implicated in molecular recognition and must thus be saturated. This requirement also applies to different binding modes of a given MCL. Moreover, both SCLs and MCLs were overall hydrophilic in nature, as indicated by low LogP values (*p*-value = $1.9\cdot 10^{-2}$). For SCLs, the median LogP was 3.2 and for MCLs, the median was 2.3. We also compared the LogP distribution with a random sample of 450 qualifying X-ray ligands according to Figure 1 without multitarget activity. The LogP median value of the random sample (3.5) differed only slightly from SCLs (3.17) but further increased compared to MCLs (2.3). Considering the complete distribution, random X-ray ligands without multi-target activity differed significantly from SCLs and MCLs (Mann–Whitney U test, SCL: *p*-value = $2.2\cdot 10^{-6}$, MCL: *p*-value = $2.8\cdot 10^{-5}$). However, it must also be taken into consideration that the *p*-value depends on sample size and provides no indication of the magnitude of the effect. In addition, a number of MCLs had LogP values approaching 0. Thus, promiscuous ligands were far from being hydrophobic in nature, as is frequently assumed. Instead, SCLs and MCLs were predominantly hydrophilic. This was another interesting finding.

**Figure 6:**
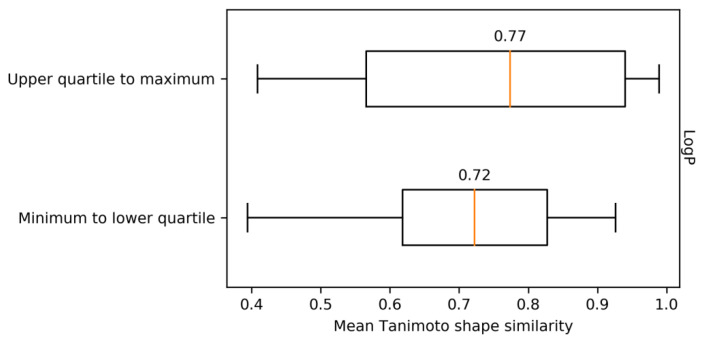
Shape similarity versus lipophilicity. For the 18 most lipophilic MCLs (25%; upper quartile to maximum LogP in Figure 5) and the 18 most hydrophilic MCLs (25%; minimum LogP to lower quartile), the distributions of mean pairwise shape similarity values are reported in boxplots. The orange line indicates the median value of each distribution.

Because the number of hydrogen bond donors, number of hydrogen bond acceptors, and LogP are correlated, a principal component analysis was also carried out to generate an orthogonal feature space for comparing SCLs and MCLs, as shown in Supplementary Figure S4. Although the first two components of the reduced feature space explained over 80% of the data variance, no further separation between SCLs and MCLs was observed, which also illustrated their global property resemblance. Figure 6 compares the mean shape similarity for the 18 most hydrophobic MCLs (25%; upper quartile to maximum LogP in Figure 5) and 18 most hydrophilic MCLs (25%; minimum LogP to lower quartile). The mean shape similarity per ligand was overall only slightly higher for more hydrophobic than hydrophilic MCLs. However, given the small sample sizes, this observation is not statistically significant (Mann–Whitney U test: *p*-value = 0.23). Hence, mean shape similarity was considered comparable for hydrophobic and hydrophilic MCLs.

### 2.6. Representative Binding Modes

**Figure 7:**
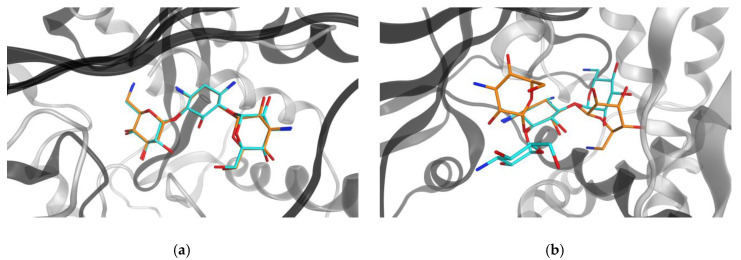
Kanamycin bound to different targets. Compared are binding modes of kanamycin in the active sites of (**a**) aminoglycoside 3’-phosphotransferase (gray, PDB ID: 1L8T), with ligand carbon atoms (LCs) colored in orange, and aminoglycoside acetyltransferase (black, PDB ID: 6BFH), LCs in cyan (shape similarity: 0.94), and (**b**) ribosome inactivating protein (gray, PDB ID: 3U6T), LCs in orange, and 2’’-aminoglycoside nucleotidyltransferase (black, PDB ID: 4WQL), LCs in cyan (shape similarity: 0.61).

**Figure 8:**
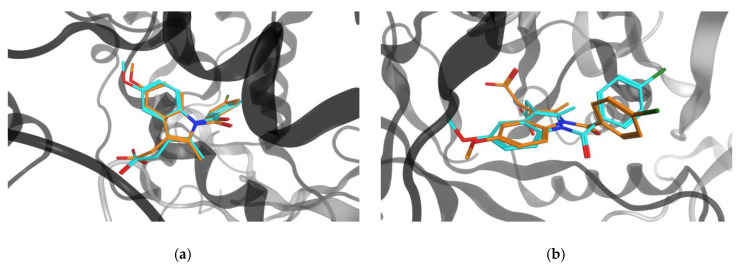
Indomethacin bound to different targets. Compared are binding modes of indomethacin in lactoylglutathione lyase (gray, PDB ID: 4KYK; LCs orange) and (**a**) prostaglandin G/H synthase 2 (black, PDB ID: 4COX; LCs cyan, shape similarity: 0.94) or (**b**) aldo-keto reductase 1_C2 (black, PDB ID: 4JQ4; LCs cyan, shape similarity: 0.68).

Figure 7 and Figure 8 compare different binding modes for two MCLs, kanamycin, an antibiotic, and indomethacin, an anti-inflammatory, respectively. The representations illustrate that both MCLs were capable of adopting very similar or different conformations when binding to target proteins from different classes, which represented a frequent binding characteristic of MCLs. PDB IDs for complexes with both ligands are reported in Supplementary Table S3.

## 3. Discussion

In pharmaceutical research, multitarget activity of small molecules is of high relevance, for more than one reason. While it provides the foundation of desirable polypharmacology, multitarget activity is also responsible for unwanted side effects of drugs. Thus, achieving a reasonable balance between desired therapeutic and undesired side effects is key for advancing promiscuous compounds. Although such compounds are intensely investigated in drug discovery, it is currently only little understood how multitarget activity of small molecules is enabled at the molecular level of detail. A deeper understanding of structural features or molecular properties that contribute to or drive multitarget activity would substantially aid in designing compounds with pre-defined activity profiles, which currently is a topical issue in pharmaceutical research. For rationalizing molecular origins of promiscuity, identifying multitarget ligands on the basis of complex X-ray structures principally is an attractive approach. Although structural data are still limited, despite significant growth over the past decade, the intrinsic advantage of basing promiscuity analysis on structural analysis is that investigated binding events are confirmed and not affected by false-positive assay artifacts. Previous studies have shown that ligands binding to members of different protein families frequently adopted similar binding modes but displayed different interaction patterns in binding sites [14,19]. These findings also raised the question of whether there might be a limited repertoire of binding modes for promiscuous ligands, which partly inspired our current study. Moreover, we were interested in exploring and characterizing promiscuous compounds capable of binding to structurally and functionally distinct proteins. Such ligands were thought to represent the most prominent cases of chemical entities with defined multitarget activity. Therefore, we designed and implemented a rigorous analysis scheme combining the exploration of structural data with chemoinformatics, as reported herein. On the basis of our analysis, we identified 70 MCLs that were widely distributed over 19 functional protein classes and compared their binding modes in different structural environments. Our study produced some unexpected results. While we also identified similar binding modes of MCLs, we found that a given MCL was often capable of binding with either very similar or very different conformations, depending on the target proteins. These results substantially extended earlier findings. In fact, binding shape diversity was a general characteristic of newly identified MCLs (and canonical binding shapes were not detected). Furthermore, we determined that MCLs and SCLs were comparably flexible and predominantly hydrophilic, another new finding. The most hydrophilic and most hydrophobic MCLs had comparable mean shape similarity.

Taken together, the results of our investigation revealed characteristic features of promiscuous small molecules binding to proteins from different classes. On the basis of our findings, the ability to adopt very similar or distinct binding modes, depending on the target proteins, is a hallmark of MCLs, consistent with their intrinsic flexibility.

## 4. Materials and Methods

### 4.1. X-ray Structures of Ligand-Target Complexes

Structures of ligand-target complexes were systematically extracted from the Research Collaboratory for Structural Bioinformatics (RCSB) Protein Data Bank (PDB) (accessed 2 March 2020) [21]. For each PDB entry, corresponding UniProt IDs [22] were mapped using BioServices (accessed 2 March 2020) [23]. Each protein was assigned to a specific class according to the GO organization scheme [20]. Unclassified proteins and others associated with multiple classes were discarded.

### 4.2. Characterization of Crystallographic Ligands and Activity Data

Information about crystallographic ligands was extracted from ‘Macromolecular Crystallographic Information Files’ (MMCIF) available in the Protein Data Bank in Europe (PDBe) (accessed March 2020), a resource integrating structural and functional data [24]. Ligands denoted as ‘obsolete’ were discarded. In addition, peptides, saccharides, nucleosides, nucleoside analogs, metal complexes, and polymers were omitted. In addition, solvent and buffer molecules were eliminated using the ChEBI database [25]. In this analysis, ligands were required to have a molecular weight (MW) between 300 and 900 Da and at least one numerically defined potency annotation of at least 10 µM (pK_i_, pK_d_, pIC_50_ ≥ 5) reported in ChEMBL (version 26) [26] or PDBbind (version 2019) [27]. On the basis of these criteria, 8331 qualifying ligands were identified and for these compounds, SMILES representations [28] were generated and standardized with the aid of the OpenEye chemistry toolkit [29]. For ligands, MW, the number of hydrogen-bond acceptors (HBA), number of hydrogen-bond donors (HBD), number of rotatable bonds, and the logarithm of the octanol-water partition coefficient (LogP) were calculated from SMILES using the RDKit [30].

### 4.3. Analysis of Ligand–Target Interactions

To restrict the analysis to qualifying compounds forming extensive ligand–target interactions, complexes with less than eight residues within 5.0 Å distance to ligand atoms were disregarded. The 5.0 Å distance was chosen because it exceeds the contact distance for weakest non-bonded polar and van der Waals interactions (~4.5 Å), and hence represents a meaningful threshold distance for intermolecular contacts. Complexes with fewer than eight residues in proximity to the ligand were disregarded because the residue count exceeded 1.5-fold of the interquartile range below the first quartile in the distribution of contact residues of X-ray ligands. Hence, these complexes represented statistical outliers. In addition, only non-covalently bound ligands were considered. On the basis of these criteria, 5707 structures of complexes remained that contained a total of 4689 ligands.

To further examine MCL assignments, systematic structural comparisons of ligand binding domains of targets of each MCL were carried out to identify conserved binding domains that might occur in proteins from different classes. After superposition, ligand binding domains of all complexes with RMSD values less than 10 Å were subjected to visual inspection and binding domains that closely superposed were identified. If a conserved binding domain was detected, only one of these targets was retained, which reduced the number of MCLs to a final count of 70.

### 4.4. Binding Mode Comparison

Bound conformations of SCLs and MCLs were extracted as structure-data files (SDF) [31] from RCSB structures. Pairwise Tanimoto shape similarity of 515 MTLs was calculated on the basis of optimally superimposed conformers using Rapid Overlay of Chemical Structures (ROCS) [29], as implemented in the OpenEye toolkit. For shape similarity calculations, molecular surface property values such as electrostatic potential were not taken into consideration in order to focus the analysis on binding shapes. In addition, RMSD of alpha carbon positions of protein structures with shared MCLs was calculated using Molecular Operating Environment (MOE) version 2019 [32]. Protein binding site similarities were calculated using SiteEngine [33].

## 5. Conclusions

Herein, we have reported a large-scale data analysis designed to identify promiscuous compounds binding to different protein classes. The set of MCLs we newly identified was thoroughly characterized focusing on binding modes and molecular properties in comparison to SCLs, which we also assembled. MCLs were shown to be mostly flexible and hydrophilic in nature. Shape diversity of binding modes in different protein environments emerged as a signature of MCLs, which provided insights into binding characteristics and molecular mechanisms underlying multitarget activity. Our findings also provide some guidance for the design of new multitarget ligands since MCLs adopting diverse binding modes can also be considered as templates for design efforts. Therefore, both MCLs and SCLs we identified in our study are freely available upon request.

## Figures and Tables

**Table 1:** MCL complexes. X-ray structures of complexes with MCLs are organized by protein class. For each class, the number of complexes, unique MCLs, and unique target proteins is reported.

Protein Class	Complexes	MCLs	Proteins
Enzyme regulator	1	1	1
Hydrolase (C-N bonds, no peptides)	32	9	31
Hydrolase (acid anhydrides)	3	2	3
Hydrolase (ester bonds)	17	13	11
Hydrolase (glycosyl bonds)	9	7	8
Isomerase	1	1	1
Ligase	2	1	2
Lyase	13	8	9
Oxidoreductase	81	36	59
Peptidase	11	8	10
Signaling receptor	7	4	5
Transcription regulator	19	14	10
Transferase (acyl groups)	12	7	10
Transferase (alkyl or aryl groups, no methyl)	28	6	21
Transferase (glycosyl groups)	11	7	9
Transferase (one-carbon groups)	22	8	16
Transferase (other)	7	5	7
Transferase (phosphorus-containing groups)	33	18	23
Transporter	22	15	19

## Supplementary Materials

The following are available online at https://www.mdpi.com/1422-0067/21/11/3782/s1, Figure S1: X-ray structure count per ligand, Table S1: Multiclass ligands, Table S2: SCL complexes, Figure S2: Distribution of rotatable bonds of MCLs in compared X-ray structures, Figure S3: Tanimoto shape similarity versus pocket similarity scores, Figure S4: Principal component analysis of the feature space, Table S3: PDB IDs of unique ligand-protein complexes with kanamycin or indomethacin.

## Abbreviations

GO	Gene Ontology
LC	Ligand carbon atom
MCL	Multiclass ligands
MTL	Multitarget ligands
PDB	Protein Data Bank
PDBe	Protein Data Bank in Europe
RMSD	Root mean square deviation
SCL	Single-class ligands
