# Supplementary material for: X-ray Structure-Based Chemoinformatic Analysis Identifies Promiscuous Ligands Binding to Proteins from Different Classes with Varying Shapes
Source: Int J Mol Sci. 2020 May 27;21(11):3782. doi: 10.3390/ijms21113782 (PMC7312685; doi:10.3390/ijms21113782)
Supplement: Supplementary file 1 [file ijms-21-03782-s001.pdf]

Supplementary Material

X-ray structure-based chemoinformatic analysis identifies promiscuous ligands binding to proteins from different classes with varying shapes

Christian Feldmann [1] and Jürgen Bajorath [1,*]

[1] Department of Life Science Informatics, B-IT, LIMES Program Unit Chemical Biology and Medicinal Chemistry, Rheinische Friedrich-Wilhelms-Universität, Endenicher Allee 19c, D-53115 Bonn, Germany; bajorath@bit.uni-bonn.de.

* Correspondence: bajorath@bit.uni-bonn.de.

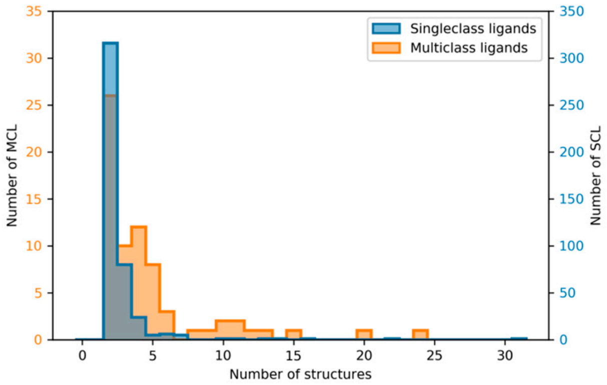

Supplementary Figure S1. X-ray structure count per ligand. The histogram reports the number of X-ray structures in which singleclass and multiclass ligands were found.

Supplementary Table S1. Multiclass Ligands. Given are PDB IDs of MCLs, the number of unique proteins with which they formed complex structures, and the corresponding number of protein classes.

Ligand-ID	#Proteins	#Protein classes
CB3	4	2
GDS	4	3
H1S	4	3
9CS	4	2
VDM	5	2
NMY	3	3
KAN	11	5
GRG	6	2
FPP	12	2
FPS	11	2
CE3	5	3
CLS	2	2
PLO	2	2
STR	10	2
HXA	2	2
6T2	2	2
AIC	5	3
REA	3	2
LP5	4	3
BB2	24	2
BFQ	4	2
4GA	2	2
4PT	2	2
IBI	2	2
R78	4	2
ERY	6	4
ET	4	2
1QK	2	2
K25	2	2
CVI	3	3
B3N	3	2
MTX	20	4
SMD	3	2
QI9	2	2
SLX	2	2
BER	5	3
NIM	2	2
MHI	2	2
8UX	2	2
TCH	3	2
CHD	5	4
CLR	6	2
FUA	3	2
DM2	2	2
198	2	2
P6U	2	2
CBW	2	2
C2F	10	2
TDZ	3	2
RFP	2	2
IMN	8	3
TPS	3	2
DEQ	3	2
SI5	2	2
I3P	9	3

4IP	4	3
K32	2	2
HD2	2	2
MYC	5	4
REF	2	2
8GQ	2	2
QUE	15	6
PNT	2	2
FFO	4	2
DHF	5	2
FOL	13	4
LYA	5	3
8PR	4	3
INR	2	2
ZST	4	2

Supplementary Table S2. SCL complexes. X-ray structures of complexes with SCLs are organized by protein class. For each class, the number of complexes, unique SCLs, and unique target proteins is reported.

Protein class	Complexes	SCLs	Proteins
Enzyme regulator	5	2	5
Hydrolase (C-N bonds, no peptides)	24	10	17
Hydrolase (acid anhydrides)	4	2	3
Hydrolase (ester bonds)	50	21	28
Hydrolase (glycosyl bonds)	78	18	55
Hydrolase (other)	4	2	2
Isomerase	20	4	16
Ligase	8	4	5
Lyase	39	18	16
Oxidoreductase	311	125	73
Peptidase	164	63	84
Signaling receptor	2	1	2
Transcription regulator	4	2	2
Transferase (acyl groups)	18	5	16
Transferase (alkyl or aryl groups, no methyl)	50	19	26
Transferase (glycosyl groups)	27	12	12
Transferase (one-carbon groups)	18	6	13
Transferase (other)	7	3	7
Transferase (phosphorus-containing groups)	337	121	142
Transporter	11	5	9

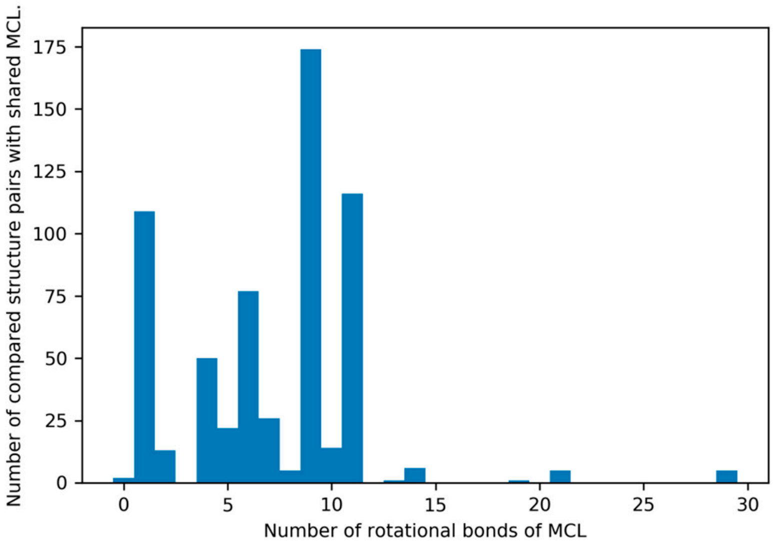

Supplementary Figure S2. Distribution of rotatable bonds of MCLs in compared X-ray structures. For X-ray structures with shared MCLs, the number of rotational bonds of MCLs is reported.

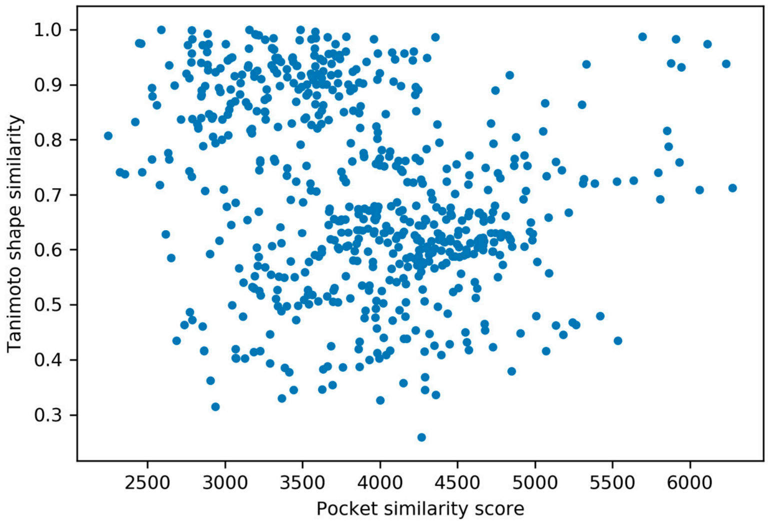

Supplementary Figure S3. Tanimoto shape similarity versus pocket similarity scores. Reported are pairwise comparisons of MCL complexes. Each dot represents a pair of complex structures with a shared MCL. Ligand shape similarity is plotted against the binding site similarity score calculated with SiteEngine.

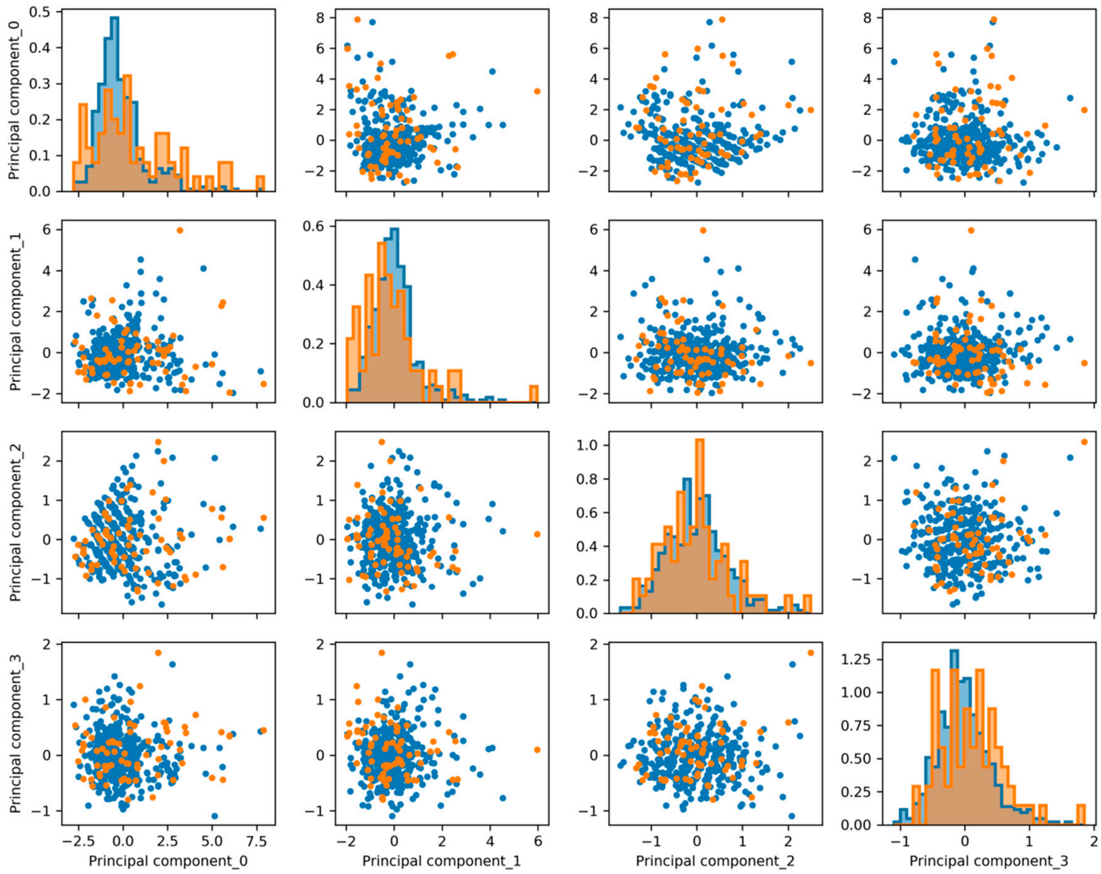

Supplementary Figure S4. Principal component analysis of the feature space. Descriptors of MCLs and SCLs were standardized and principal component analysis of the multivariate feature space was carried out with scikit-learn. Component 0 represented 60.6% of explained data variance while components 1, 2, and 3 accounted for 23.8 %, 11.1%, and 4.5% of explained variance, respectively. MCLs (blue) and SCLs (orange) are represented as points in pair-wise component space. Diagonal plots represent the distribution of component values as histograms.

Supplementary Table S3. PDB IDs of unique ligand-protein complexes with kanamycin or indomethacin.

Kanamycin	Indomethacin
1L8T	1S2A
1M4I	2DM6
1ND4	2OTH
3KP5	2ZB8
3U6T	3OGW
4EM0	4COX
4FEU	4JQ4
4OKN	4KYK
4WQL	
6BFH	
6O5U	
11 Proteins	8 Proteins
5 Protein classes	3 Protein classes
